# Supplementary material for: Comparative expression profiling reveals widespread coordinated evolution of gene expression across eukaryotes
Source: Nat Commun. 2018 Nov 23;9:4963. doi: 10.1038/s41467-018-07436-y (PMC6251915; doi:10.1038/s41467-018-07436-y)
Supplement: Supplementary file 1 — Supplementary Information [file 41467_2018_7436_MOESM1_ESM.pdf]

Supplementary Information

Comparative expression profiling reveals widespread coordinated evolution of gene expression across eukaryotes

Trevor Martin and Hunter B. Fraser

Supplementary Figures

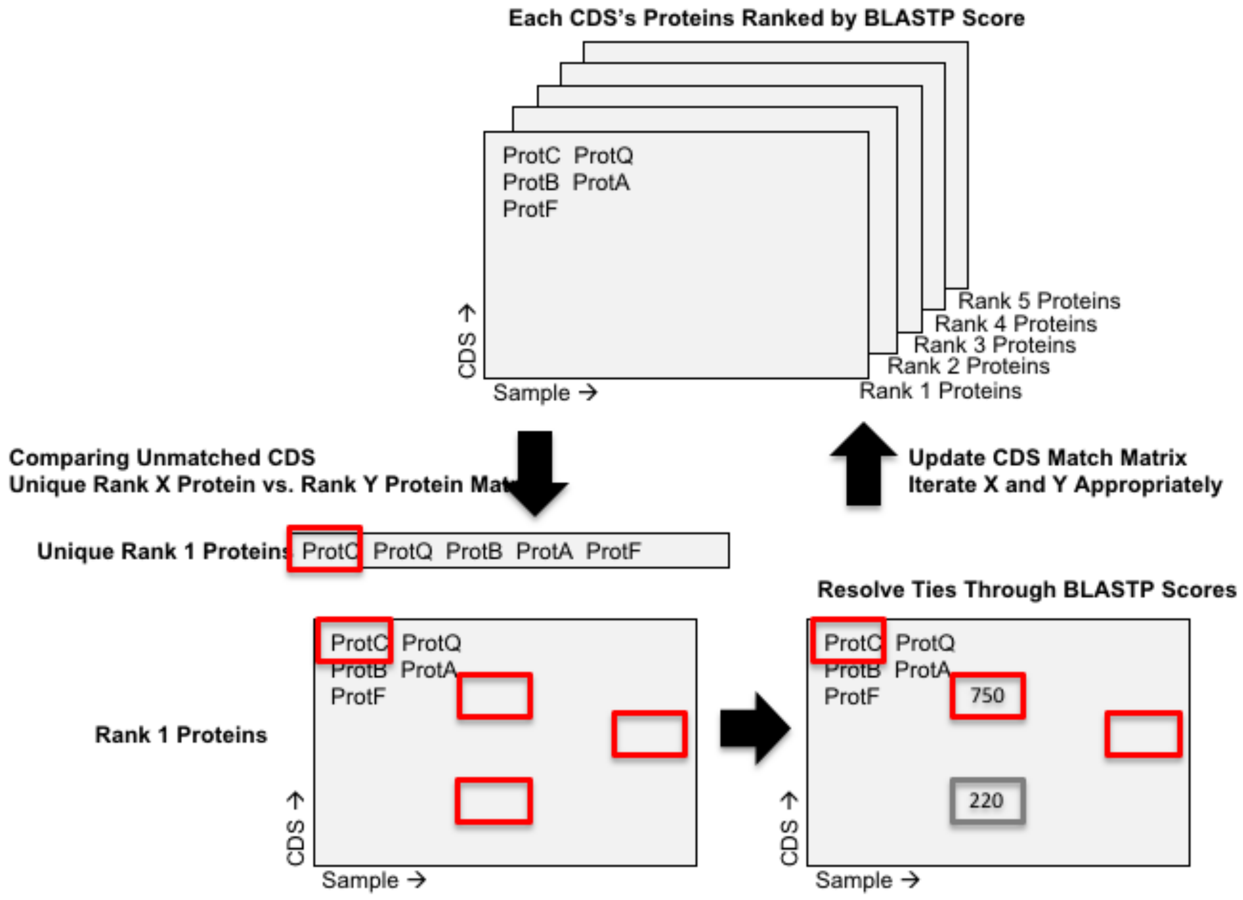

Supplementary Figure 1: **Combining transcripts into ortholog groups.**

Each sample has a set of coding sequences (CDS) with measured expression and up to five UniRef IDs identified and ranked by BLASTP score. To begin, all the unique rank one UniRef IDs are used to create a vector of expression for an ortholog group by matching each rank one UniRef ID to the samples with that ID that are also rank one. This process is then repeated by iterating through each set of ranked proteins until all expression values are matched.

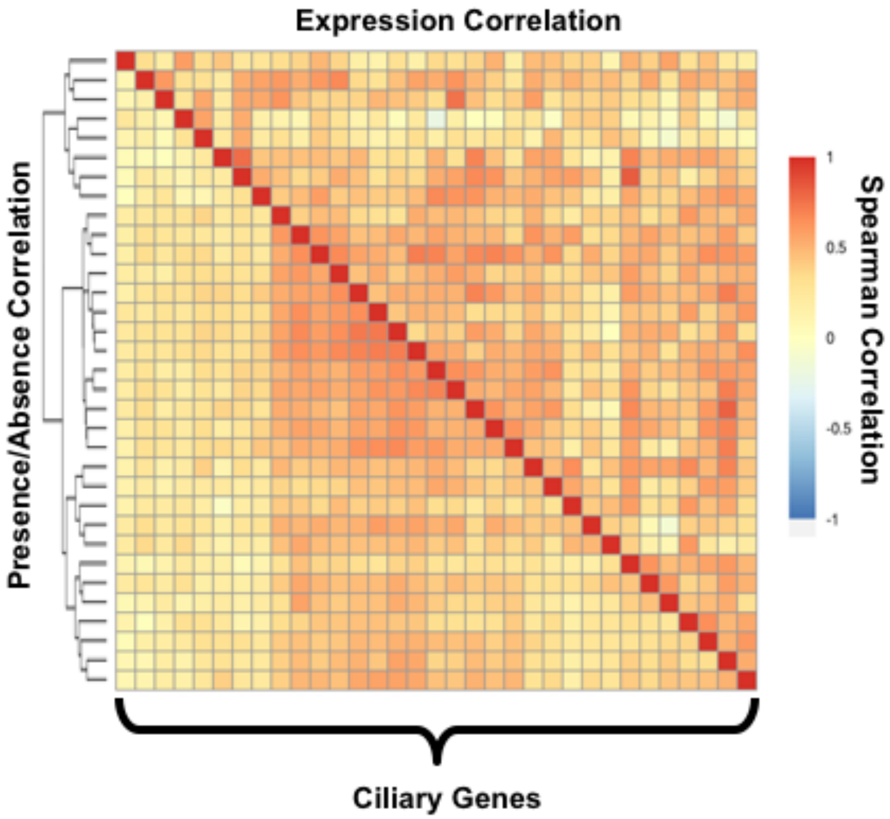

Supplementary Figure 2: **Full heatmap for PP and PEP correlations among ciliary genes.** Heatmap of the PP (gene presence/absence, bottom left) and PEP (expression, top right) Spearman correlation-based scores (see Methods) among ciliary genes. Both axes are hierarchically clustered by the Euclidean distance between the gene PP scores.

Aminoacyl-tRNA Biosynthesis Gene Set in CLIME:

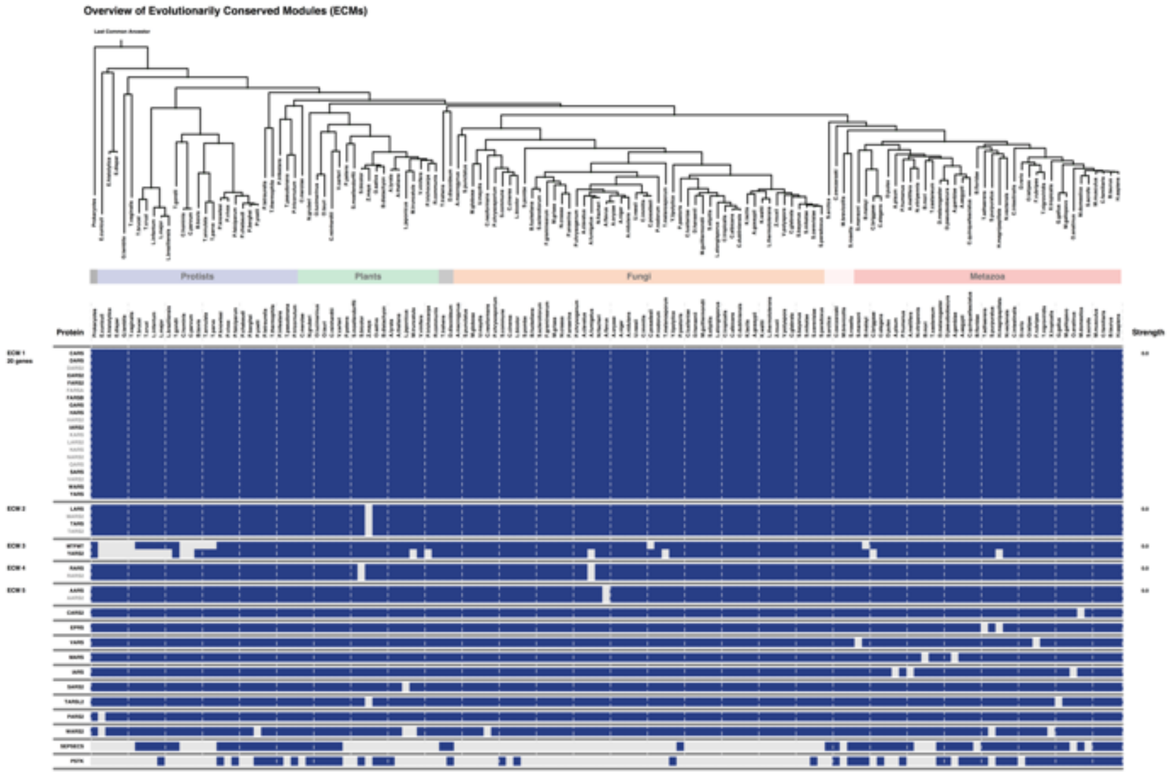

Mismatch Repair Gene Set in CLIME:

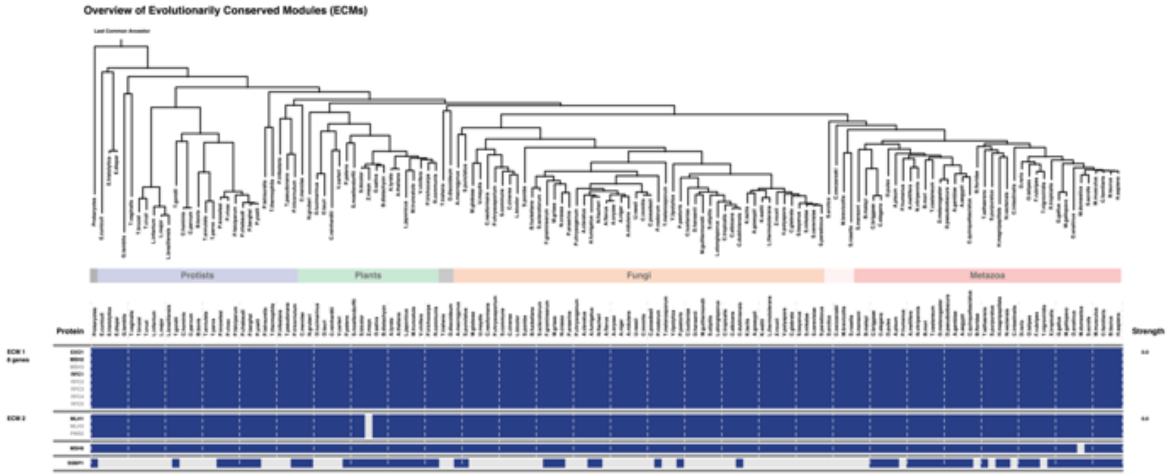

Present Absent

Supplementary Figure 3: Coordinated evolution between gene sets and addition of novel genes to known gene sets.

(A) The coordinated evolution scores between the gene sets for the Golgi apparatus and genes downregulated in Alzheimer's disease is shown as a heatmap with the same scale as in (B). The gene pair highlighted in green is shown as a scatterplot to the right; each point is a sample with measured expression. **(B)** The coordinated evolution scores for diabetes pathway genes are shown in heatmap form. In green are the two genes not in this gene set with the strongest PEP scores to the known genes in this set.

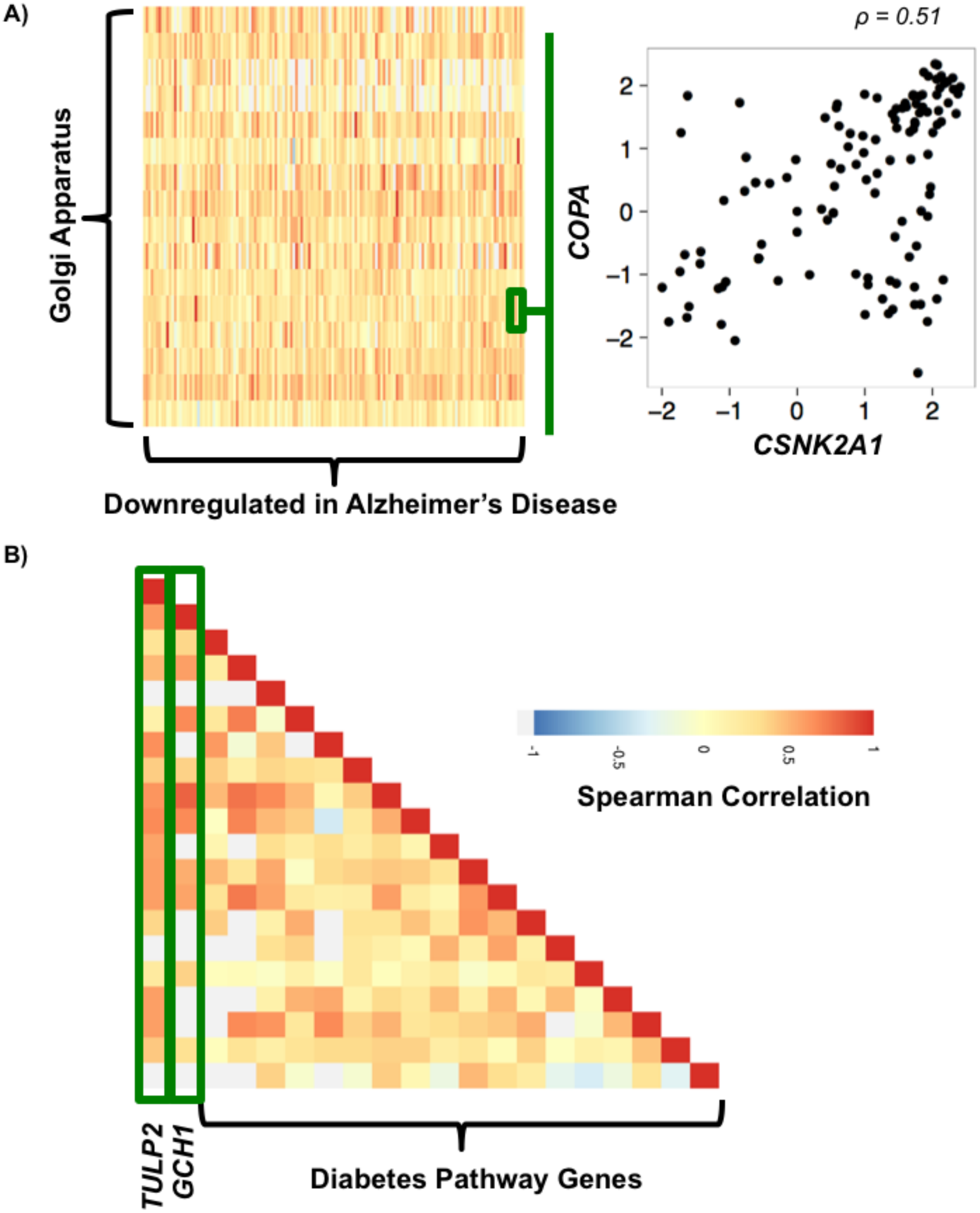

Supplementary Figure 4: **Genes unlikely to be lost are hidden from phylogenetic profiling.** Two of the gene sets identified in this study, aminoacyl-tRNA biosynthesis and mismatch repair were also analyzed in a recent PP study[6] and their patterns of loss over evolutionary time are

shown here (blue is gene sequence presence and gray is absence). Neither of these gene sets results in informative signs of correlated loss.
