# Supplementary material for: Comparative expression profiling reveals widespread coordinated evolution of gene expression across eukaryotes
Source: Nat Commun. 2018 Nov 23;9:4963. doi: 10.1038/s41467-018-07436-y (PMC6251915; doi:10.1038/s41467-018-07436-y)
Supplement: Supplementary file 3 — Description of Additional Supplementary Files [file 41467_2018_7436_MOESM3_ESM.pdf]

Supplementary Tables

Supplementary Table 1: Full MMETSP gene expression matrix.

Supplementary Table 2: Gene sets with PEP coordinated evolution at a 5% FDR.

Supplementary Table 3: Gene set pairs with significant PEP coordinated evolution.
